# Clinicopathological study of glomerular diseases associated with sarcoidosis: a multicenter study

**DOI:** 10.1186/1750-1172-8-65

**Published:** 2013-04-30

**Authors:** Thomas Stehlé, Dominique Joly, Philippe Vanhille, Jean-Jacques Boffa, Philippe Rémy, Laurent Mesnard, Maxime Hoffmann, Philippe Grimbert, Gabriel Choukroun, François Vrtovsnik, Jérôme Verine, Dominique Desvaux, Francine Walker, Philippe Lang, Matthieu Mahevas, Dil Sahali, Vincent Audard

**Affiliations:** 1 Service de Néphrologie et Transplantation, Hôpital Henri Mondor, Centre de référence maladie rare Syndrome Néphrotique Idiopathique, Institut Francilien de recherche en Néphrologie et Transplantation (IFRNT), INSERM U955, Université Paris Est Créteil, APHP (Assistance Publique–Hôpitaux de Paris, Créteil), Créteil, France; 2 Service de Néphrologie, Hôpital Necker, Université Paris Descartes, INSERM U845, APHP, Paris, France; 3 Service de Néphrologie Hôpital de Valenciennes, Valenciennes, France; 4 Service de Néphrologie, Hôpital Tenon, APHP, INSERM UMR S 702, UPMC (Université Pierre et Marie Curie), Paris, France; 5 Urgences Néphrologiques & Transplantation Rénale, Hôpital Tenon, APHP, INSERM UMR S 702, UPMC Université Paris 06, Paris, France; 6 Néphrologie Hémodialyse, Hôpital Privé La Louvière, Lille, France; 7 Service de Néphrologie, Médecine Interne, Dialyse, Transplantation et Réanimation Médicale, Centre Hospitalier Universitaire d'Amiens, Hôpital Sud, Amiens, France; 8 Service de Néphrologie, Hôpital Bichat, APHP, Université Paris Diderot, Paris, France; 9 Service de Pathologie, Hôpital Saint Louis, APHP, Université Paris Diderot, INSERM U728, Paris, France; 10 Département de Pathologie, Hôpital Henri Mondor, Institut Francilien de recherche en Néphrologie et transplantation (IFRNT), INSERM U955, Paris Est Université, APHP, Créteil, F-94010, France; 11 Service d’Anatomopathologie, Hôpital Bichat, APHP, Université Paris Diderot, Paris, F-75018, France; 12 Service de Médecine Interne, Centre de référence maladie rare des cytopénies auto-immunes de l’adulte, Hôpital Henri Mondor, APHP, Université Paris Est Créteil, Créteil, France

**Keywords:** Nephrotic syndrome, Glomerular disease, Kidney biopsy, Sarcoidosis

## Abstract

**Background:**

The association between sarcoidosis and glomerular diseases has not been extensively investigated in a large series and the potential features of this uncommon association remain to be determined.

**Methods:**

We retrospectively identified 26 patients with biopsy-proven glomerular lesions that occurred in a sarcoidosis context. Potential remission of glomerular disease and sarcoidosis under specific treatment (steroid and/or immunosuppressive agents) was recorded for all patients. Demographic, clinical and biological characteristics were assessed at the time of kidney biopsy for each patient. Therapeutic data were analyzed for all patients.

**Results:**

Glomerular disease occurred after the diagnosis of sarcoidosis in 11 of 26 cases (42%) (mean delay of 9.7 years). In six patients (23%), the glomerulopathy preceded the sarcoidosis diagnosis (mean delay 8 years). In the last nine patients (35%), both conditions occurred simultaneously. The most frequent glomerular disease occurring in sarcoidosis patients was membranous nephropathy in eleven cases. Other glomerular lesions included IgA nephropathy in six cases, focal segmental glomerulosclerosis in four patients, minimal change nephrotic syndrome for three patients and proliferative lupus nephritis in two patients. Granulomatous interstitial nephritis was associated with glomerular disease in six patients and was exclusively found in patients in whom the both disease occurred simultaneously. In nine patients with simultaneous glomerular and sarcoidosis diseases, we observed a strong dissociation between glomerular disease and sarcoidosis in terms of steroid responsiveness. At the end of the follow-up (mean of 8.4 years), six patients had reached end-stage renal disease and three patients had died.

**Conclusions:**

A wide spectrum of glomerular lesions is associated with sarcoidosis. The close temporal relationship observed in some patients suggests common causative molecular mechanisms of glomerular injury but complete remission of both diseases in response to exclusive steroid therapy is infrequent.

## Background

Sarcoidosis is a chronic multisystemic inflammatory disease of unknown origin, characterized by the presence of non-caseating, epitheliod granulomas in some tissues leading to organ dysfunction [1,2]. Sarcoidosis typically affects young adults and its clinical course ranges from spontaneous resolution to chronic progressive disease. The severity and diversity of the clinical manifestations related to sarcoidosis depend on the extent of the infiltrating granulomatous lesions, which preferentially involve the lower respiratory tract. Granulomatous lesions can also affect extrapulmonary sites, such as the lymph nodes, heart, kidneys, central nervous system, liver, spleen, larynx and eyes [2,3]. Pathophysiological mechanisms involved in the formation, maintenance or spontaneous resolution of sarcoidosis granuloma thought to be linked to genetic susceptibility and to unidentified environmental antigens which trigger an uncontrolled cell-mediated immune reaction involving macrophages and CD4 type 1 helper T (Th1) cells [2,4,5].

The prevalence of kidney impairment in sarcoidosis patients ranges from 10% to 20% of cases [6]. Renal injury is most commonly due to disorders in calcium homeostasis, with renal stone disease and nephrocalcinosis [6]. Granulomatous tubulointerstitial nephritis (GTIN) is a less common cause of renal lesions that occurs in approximately 20% of patients with sarcoidosis [7], and benefits from steroid therapy [8,9]. By contrast, glomerular diseases have been rarely reported in patients with sarcoidosis [6]. Most publications reporting glomerular diseases in the context of sarcoidosis consisted exclusively in case reports. The nature of glomerular lesions seems to be diverse, with membranous nephropathy (MN) [10-14]; minimal change nephrotic syndrome (MCNS) [15-17]; focal segmental glomerulosclerosis (FSGS) [18-20], imunoglobulin A nephropathy (IgAN) [21-24] and proliferative glomerulonephritis [13,25,26].

To our knowledge no extensive study has been performed to describe the spectrum of glomerular diseases that occurs in the context of sarcoidosis**.** In this study, we retrospectively identified 26 patients with biopsy-proven glomerular disease and sarcoidosis and further analyzed the clinical, histological, laboratory, and therapeutic data of these patients to assess the significance of this rare association.

## Methods

### Patients

Twenty-six patients suffering from both sarcoidosis and glomerular disease were retrospectively identified and followed between 1977 and 2012 in the Nephrology Departments of seven French hospitals (Henri Mondor Hospital, Necker Hospital, Valenciennes Hospital, Tenon Hospital, La Louvière Hospital, Amiens Hospital, Bichat Hospital). We conducted this retrospective study by sending a questionnaire to all nephrology departments involved in the management of glomerular diseases to determine if some patients exhibited glomerular involvement in the context of sarcoidosis occurrence. In each hospital, patients were identified by computing of the renal pathology and clinical diagnosis databases. All patients underwent a renal biopsy for the exploration of proteinuria and/or renal impairment. Demographic, clinical and laboratory data were assessed for each patient at the time of kidney biopsy. Glomerular kidney disease was considered as occurring simultaneously with sarcoidosis when the delay between the diagnoses of both diseases was less than three months. Follow up data (at least six months after kidney biopsy) were obtained for all patients.

### Sarcoidosis diagnosis

Sarcoidosis was diagnosed according to the statement of the American Thoracic Society, the European Respiratory Society and the World Association of Sarcoidosis [27]. Its diagnosis was supported by a body of evidence including, clinical, biological, radiographic findings and/or histological evidence of non-caseating granulomas on tissue biopsy [1,2,5]. The number of affected organs was systematically reviewed for all patients. In patients with lung involvement, the four-stage radiographic classification of sarcoidosis was used to determine the severity of the pulmonary lesions associated with sarcoidosis: stage 1 for bilateral hilar lymphadenopathy; stage 2 for pulmonary infiltrates associated with intrathoracic adenopathies; stage 3 for isolated parenchymal infiltration without fibrosis; and stage 4 consisted of pulmonary fibrosis [28]. Sarcoidosis therapy, including steroid and/or immunosuppreseive agents use was recorded for all patients.

### Glomerular disease diagnosis

Chronic kidney disease was defined as a permanently (lasting at least three months) decrease of estimated glomerular filtration rate (eGFR) of less than 60 ml/min/1.73 m^2^ according to the modification of diet in renal disease (MDRD) formula [29]. Proteinuria was defined as an albumin excretion rate of > 0.3 g/d. Nephrotic syndrome was defined by urinary protein excretion rates of more than 3 g/d and serum albumin excretion rates below 30 g/L.

Diagnosis of MCNS included the presence of minimal change glomerular lesions and the absence of immunoglobulin and/or complement deposits [30]. FSGS lesions were identified by the presence of segmentally collapsed glomerular capillaries with areas of glomerular scarring associated with focal and segmental granular deposition of IgM and C3 within the segmental glomerular scleroses [31]. MN was characterized granular subepithelial deposition of immunoglobulins and complement along the basement membrane in IF study [32]. Diagnosis of IgAN consisted of the presence of IgA-dominant or codominant immune deposits within the mesangium [33]. Patients who fulfilled the 1997 American College of Rheumatology revised criteria for systemic lupus erythematosus (SLE) with documented sarcoidois were also included in this study [34]. In these cases, renal pathological lesions of SLE were analyzed according to the 2003 International Society of Nephrology and Renal Pathology Society (ISN/RPS) classification [35].

For patients with a diagnosis of MN, MCNS or FSGS, a proteinuria level of less than 0.3 g/d at the six-month follow-up defined a complete remission. Patients with a proteinuria level between 0.3 and 3 g/d and those whom with at least 50% reduction in the level of proteinuria, with an albumin level of > 30 g/L were considered to be in partial remission. IgAN and lupus nephritis were considered to be in remission when the proteinuria level decreased (of more than 50% of initial proteinuria level) with the stabilization or improvement of GFR. Specific treatment of glomerular disease (steroid and/or immunosuppressive treatment) was recorded for all patients.

## Results

### Clinical and biological data of patients with glomerular disease and sarcoidosis

Twenty-six patients (18 men and 8 women) were retrospectively identified. Their demographic, clinical and biological data are summarized in Table 1. The mean ages at the onset of the sarcoidosis and glomerular disease diagnosis were 37 years (range of 19–56 years) and 39 years (range of six months-59 years), respectively. In 22 patients, the diagnosis of sarcoidosis was confirmed by organ or tissue biopsy. Pathological confirmation of the sarcoidosis diagnoses was based on lung biopsy (nine cases), skin biopsy (three cases), lymph node biopsy (seven cases), muscle biopsy (one case), peritoneal biopsy (one case), and salivary glands biopsy (one case). In four patients, the diagnosis of sarcoidosis was based only on compatible clinical, biological and radiographic presentations [27].

**Table 1:** Demographic, clinical and laboratory data of all patients with glomerular disease and sarcoidosis

**Characteristics**	**Overall population**	**MN**	**IgAN**	**MCNS**	**FSGS**	**Lupus nephritis**
Number of patients	26	11(42%)	6 (23%)	3 (12%)	4 (15%)	2 (8%)
Sex (women/men)	8/18	3/8	2/4	0/3	2/2	1/1
Mean age at sarcoidosis diagnosis (yrs) (range)	37 (19–56)	39 (19–52)	34 (22–37)	36 (29–54)	40 (28–56)	36 (35–37)
Mean age at GD diagnosis (yrs) (range)	39 (0.6-59)	40 (27–57)	38 (22–53)	29 (0.5-59)	47 (38–57)	39 (36–44)
Number of patients with both conditions occurring simultaneously (%)	9 (35%)	3	3	1	2	0
Number of patients with GD before sarcoidosis (%)	6 (23%)	5	0	1	0	0
Number of patients with GD after sarcoidosis (%)	11 (42%)	3	3	1	2	2
Mean number of organs affected by sarcoidosis (range)	2.6 (1–6)	2.3 (1–3)	3.2 (1–6)	2.7 (2–3)	2.3 (1–4)	3.5 (3–4)
Steroid therapy for sarcoidosis (n)	16 (61%)	5	4	2	3	2
Sarcoidosis controlled by steroid therapy	8	2	3	2	1	0
Steroid-dependent or -resistant sarcoidosis	8	3	1	0	2	2
Mean creatinine level (mg/dL) (range)	1.40 (0.68-3.42)	1.30 (0.68-2.99)	1.63 (0.68-2.41)	1.14 (1.02-1.27)	1.75 (1.05-3.42)	0.94 (0.84-1.04)
Mean GFR (ml/min/1.73 m^2^) (range)	70.7 (16–133)	79.2 (23–133)	60.9 (26–97)	77.1 (62–92)	51.2 (16–73)	86 (61–111)
Mean proteinuria level (g/d) (range)	5.7 (0.45-20)	7.4 (0.86-20)	5 (1–12)	3.3 (3–3.6)	4.5 (1.7-6.6)	0.7 (0.45-1)
Serum albumin (g/L)	25,9 (9.7-43)	24,3 (9.7-43)	31 (17–39)	22.3 (15–29)	23.5 (11.3-38)	38 (38–38)
Number of patients with NS	15	7	2	3	3	0
GTIN associated with GD (number)	6	2	2	1	1	0
Mean follow-up (months)	101	124	48	157	80	72
Remission of GD at the end of the follow-up						
CR	9	4	2	1	1	1
PR	7	3	2	1	1	0
Mean GFR at the end of the follow-up (ml/min/1.73 m^2^) *	67.8	68.7	76.4	68.1	48.3	66.5
End-stage renal disease during follow-up	6	2	1	1	1	1
Death during the follow-up	3	1	1	0	1	0

MN: Membranous Nephropathy, IgAN: IgA nephropathy, MCNS: minimal change nephrotic syndrome, FSGS: focal and segmental glomerulosclerosis, GD: glomerular disease, GFR: glomerular filtration rate, NS: nephrotic syndrome, GTIN: granulomatous tubulointerstitial nephritis, CR: complete remission, PR: partial remission.

* after exclusion of patients with end-stage renal disease.

Chest radiographs confirmed the stages of the sarcoidosis thoracic involvement in 22 patients: nine patients had stage 1, ten patients had stage 2 and three patients had stage 3. Extrathoracic localization (other than GTIN) was present in 22 patients. The mean number of organs affected by sarcoidosis was estimated to be 2.6.

Sarcoidosis was considered to be an indolent disease, not requiring specific curative treatment in nine patients (34%). One patient received exclusively topical steroid therapy for localized uveitis. Sixteen patients (61%) received systemic steroid therapy to control active, life-threatening sarcoidosis. In eight patients, steroid therapy led to complete remission of granulomatous disease. Among the eight remaining patients, three developed steroid-dependent sarcoidosis, which required prolonged therapy, whereas five patients displayed steroid-resistant sarcoidosis, which required immunosuppressive therapy consisting of Azathioprine (two cases) or Plaquenil (three cases).

Glomerular disease was diagnosed after sarcoidosis in 11 cases (42%) with a mean delay of 9.7 years (range ten months to 20 years) between the two diagnoses (Table 1)**.** Sarcoidosis was considered to be in complete remission at the time of renal biopsy in six of these patients, whereas it was either: indolent and not treated in three patients; resistant to steroid therapy in one patient; and steroid-dependent in one patient. In six patients (23%), the glomerulopathy preceded the diagnosis of sarcoidosis, with an average delay of eight years (range from six months to 25 years) between the two diagnoses. Nine patients (35%) simultaneously displayed glomerular disease and sarcoidosis (Table 2). All of these patients (except patient 2 [pt2]) received exclusive steroid therapy to treat both conditions, regardless of the type of glomerular lesions. With this therapeutic management, sarcoidosis was considered to be in complete remission in four cases (pt5, pt7, pt8 and pt9). In these patients, effective treatment of sarcoidosis was associated with complete remission of glomerular disease in only one case (pt7) and with partial remission in three cases (pt5, pt8 and pt9). In three other patients (pt1, pt3 and pt6), steroid therapy led to complete remission of glomerular disease but failed to induce remission of sarcoidosis. In one patient (pt4), steroids failed to induce remission of either glomerular disease or sarcoidosis.

**Table 2:** Characteristics of the eight patients with sarcoidosis and glomerular disease occurring simultaneously

	**pt1**	**pt2**	**pt3**	**pt4**	**pt5**	**pt6**	**pt7**	**pt8**	**pt9**
Type of GD	MN	MN	FSGS	FSGS	IgAN	IgAN	IgAN	MCNS	MN
Age at GD diagnosis (years)	51.9	50.6	56.4	46.3	52.5	22.2	25.2	28.7	44
Proteinuria level (g/d)	1.8	4.3	1.7	6.6	1	1.3	1	3	7
GFR (ml/min/1.73 m^2^)	35	69	56	60	97	96	81	92	89
Serum Albumin (g/L)	43	31.6	38	27	17	35	38	29	16
GTIN (+ present -absent)	+	-	+	-	+	+	-	+	+
Affected organs	Lymph nodes Erythema Nodosum	Spleen Lymph nodes parotitis	Lung Skin	Lung Lymp nodes	Lung Lymph nodes Liver Spleen Bone marrow Salivary glands	Lung Lymph nodes Liver Spleen parotidis	Lymph nodes	Lymph nodes Skin epididymitis	Lymph nodes Salivary glands
Radiographic classification (stage)	1	0	2	2	3	1	1	1	1
Steroid treatment	yes	no	yes	yes	yes	yes	yes	yes	yes
Remission of GD with steroids	CR	no	CR	no	PR	CR	CR	PR	PR
Remission of sarcoidosis with steroids	no	no	no	no	yes	no	yes	yes	yes
Other immunosuppressive treatment (date of introduction)	Plaquenil (M7)		Plaquenil (M19)	Aza (M13)		Aza (M18)		MMF (M12)	
Duration of follow-up (years)	9	0.6	2.7	5	0.6	3.3	10.8	2	0.6
GFR at the end of follow-up (mL/min/1.73 m^2^)	56.8	67	died	dialysis	86.6	120.4	78.1	80	72

GD: Glomerular disease, MN: membranous nephropathy, IgAN: IgA nephropathy, MCNS: minimal change nephrotic syndrome, FSGS: focal and segmental glomerulosclerosis, GTIN: granulomatous tubulointerstitial nephritis, GFR: glomerular filtration rate, Aza: azathioprine, MMF: Mycophenolate Mofetil, CR: complete remission, PR: partial remission.

**Figure 1:**
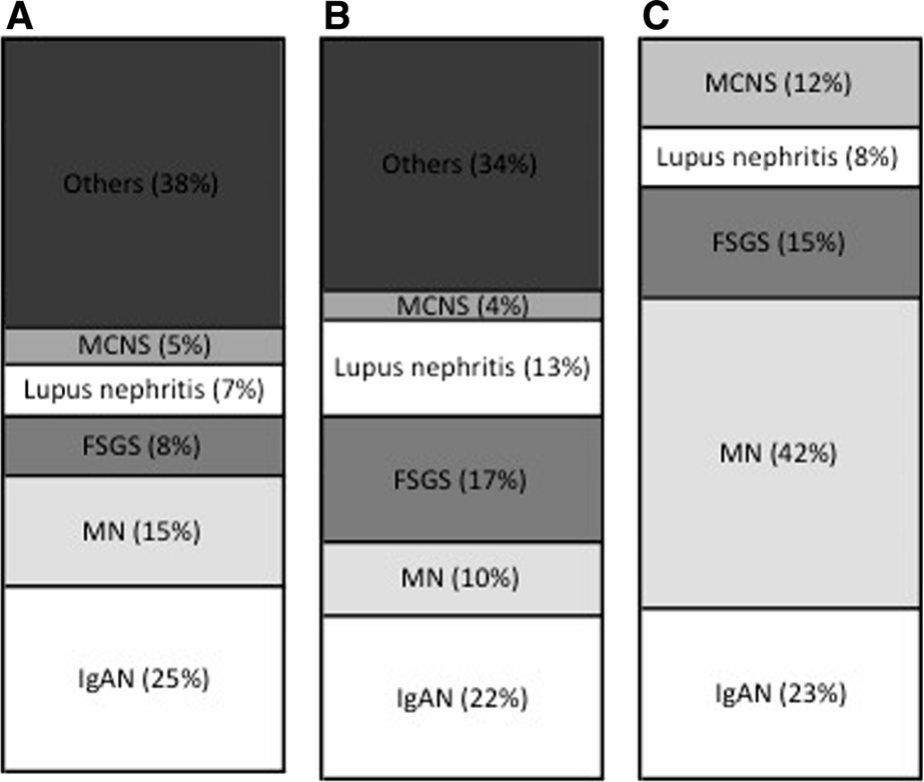
**Incidence of glomerular diseases associated with sarcoidosis compared to their distribution in general population. A**: Italian population, 11795 biopsies with glomerular diseases among 13835 biopsies, from 1987 to 1993. **B**: American population, 195 biopsies from 1974 to 2003. **C**: Study population, from 1977 to 2012. MCNS: Minimal change nephrotic syndrome, FSGS: Focal segmental glomerulosclerosis MN: Membranous nephropathy, IgAN: IgA nephropathy.

**Figure 2:**
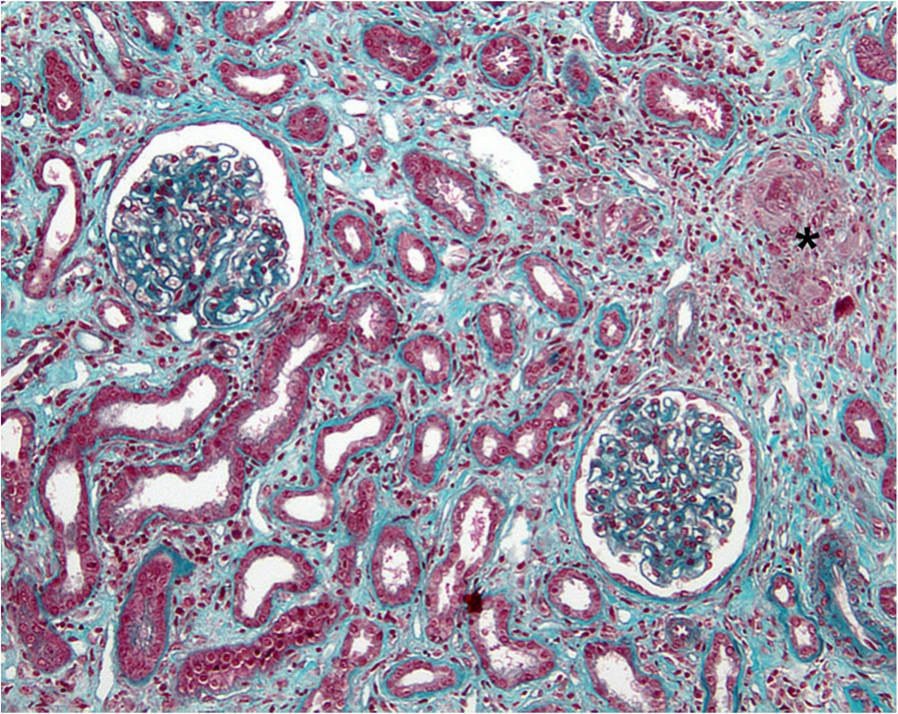
**Representative case of GTIN associated with MN (pt1) (Masson’s trichrome staining).** There is an epithelioid and giant cell granuloma adjacent to apparently normal glomeruli. The IF analysis revealed subepithelial granular deposits of IgG (data not shown).

**Figure 3:**
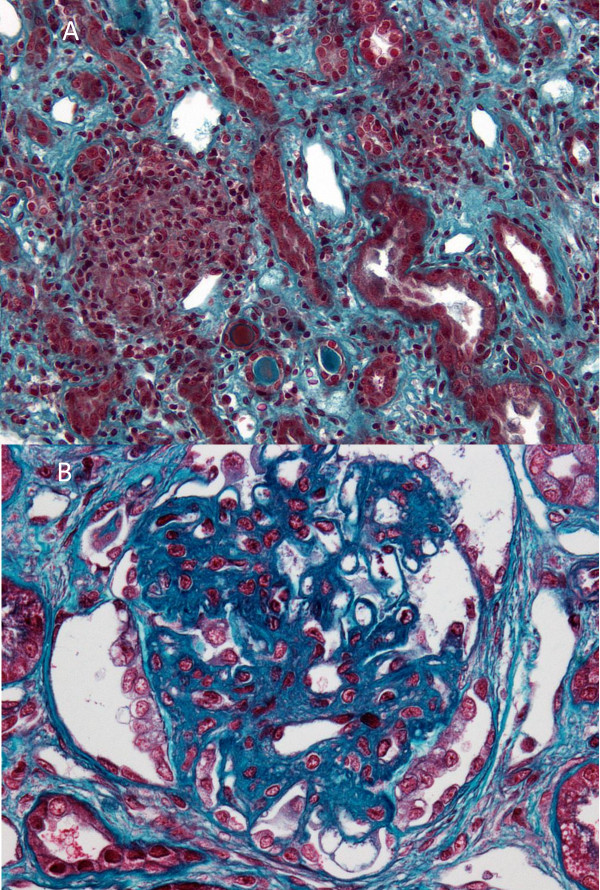
**Granulomatous interstitial nephritis occurring simultaneously with FSGS lesions (pt 3) (Masson’s trichrome staining). A**. An interstitial noncaseating epithelioid granuloma associated with lymphocytes. Some tubulitis lesions are noted. **B**. Typical FSGS lesion as a dense glomerular scar with adhesion (synechia) to Bowman’s capsule surrounded by a halo and visceral epithelial cell hyperplasia.

**Figure 4:**
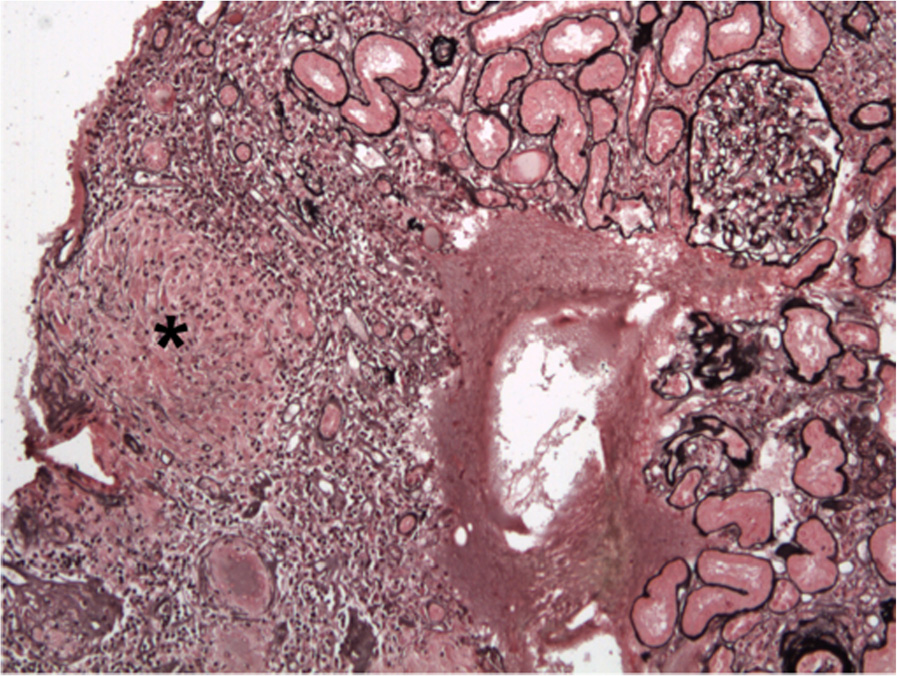
**MCNS associated with GTIN (pt8) (Jones’ silver stain).** Diffuse granuloma infiltration composed of epithelioid and giant cells throughout the interstitium without glomerular lesions in a patient with nephrotic syndrome. The IF analysis was negative for all analyzed glomeruli.

The spectrum of glomerular lesions associated with sarcoidosis consisted of MN in eleven patients (42%), IgA nephropathy in six cases (23%), FSGS in four patients (15%), MCNS for three patients (12%) and proliferative lupus nephritis in two patients (8%). As showed in Figure 1, MN seems to be more common in the context of sarcoidosis than in general population (42% of cases vs 10–15% of glomerular diseases in general population) whereas incidence of other glomerular diseases seems to be quite similar [36,37]. GTIN was observed in association with glomerular injury in six patients (23%) (Figures 2, 3A, 3B and 4). GTIN was exclusively present in renal biopsy of patients exhibiting concomitantly sarcoidosis and glomerular disease (Table 2). Mean proteinuria level at the onset of renal involvement was estimated to be 5.7 g/d (range of 0.45-20 g/d). Mean GFR at the time of glomerular disease diagnosis was 70.7 mL/min/1.73 m^2^ (range of 16–133 ml/min per 1.73 m^2^). Nine patients displayed significant renal impairment (GFR of < 60 ml/min/1.73 m^2^) at the time of renal biopsy.

### Membranous nephropathy

In our study, MN was the most frequent glomerular disorder associated with sarcoidosis (42% of cases). Among these cases, one of them has been described previously [14]. In this group, only three patients exhibited glomerular lesions simultaneously with sarcoidosis, whereas MN preceded the onset of sarcoidosis in five patients (mean delay of 4.5 years). In three other patients, MN was diagnosed after sarcoidosis (mean delay of 12.6 years). Mean proteinuria level was 7.4 g/d and the mean GFR at the time of MN diagnosis was estimated to be 79.2 mL/min/1.73 m^2^. In two patients with simultaneous MN and sarcoidosis (pt1 and 9), the renal biopsy revealed a typical GTIN pattern in association with glomerular injury. In patient 1, steroid therapy induced complete remission of MN, but did not induce remission of sarcoidosis until after Plaquenil treatment was added to steroid therapy (Table 2).

### IgA nephropathy

Among the six patients with biopsy-proven IgAN, three of them presented with glomerular disease and sarcoidosis simultaneously. In three other patients, IgAN occurred after sarcoidosis with a mean delay of 6.7 years. The mean proteinuria level at presentation was 5 g/day. The mean GFR of this subgroup of patients was estimated at 60.9 mL/min/1.73 m^2^. All patients with simultaneous sarcoidosis and IgAN (pt5, pt6 and pt7) received steroid treatment, which led to complete remission of sarcoidosis in two cases (pt 5 and pt7) (Table 2), whereas Azathioprin treatment was required for uncontrolled sarcoidosis in one patient (pt6). Steroid therapy induced complete remission of IgAN in two of these three cases (pt6 and pt7) and partial remission in one case (pt5).

### Focal segmental glomerulosclerosis

Four patients exhibited typical FSGS lesions on renal biopsy. Renal function was significantly impaired (mean GFR of 51.2 mL/min/1.73 m^2^) and the mean proteinuria level was 4.5 g/d. Two patients were diagnosed with FSGS and sarcoidosis simultaneously and two patients developed FSGS after sarcoidosis with delays of 7.3 years and 19.8 years. Typical GTIN lesions were also found in the biopsy of one patient who had simultaneous FSGS and sarcoidosis (pt3). Steroid therapy for this patient (pt3) led to complete remission of proteinuria but did not significantly improve sarcoidosis. In the second patient with simultaneous FSGS and sarcoidosis (pt4), steroid treatment failed to reduce proteinuria or to improve sarcoidosis symptoms. Despite Azathioprin treatment, sarcoidosis remained uncontrolled and the patient progressed to end-stage renal disease 3.7 years after initial presentation.

### Minimal change nephrotic syndrome

Three patients exhibited MCNS in the context of sarcoidosis. In one case, both diseases developed simultaneously whereas one patient had MCNS 5 years after sarcoidosis and one acquired it 25 years before the onset of sarcoidosis. Nephrotic syndrome without significant impairment of renal function was present in all patients at the time of renal biopsy (mean GFR: 77.1 ml/min per 1.73 m^2^). GTIN was associated with MCNS lesions in the patient with concomitant MCNS and sarcoidosis (pt8). In this patient, steroid and mycophenolate mofetyl therapies led to partial remission of the nephrotic syndrome, whereas sarcoidosis was considered to be in complete remission after steroid therapy. The patient who acquired MCNS before sarcoidosis had multiple relapses of nephrotic syndrome and received several lines of immunosuppressive treatment for steroid-dependent MCNS. Unexpectedly, steroid and/or immunosuppressive treatments were not administered at the time sarcoidosis was diagnosed. Re-introduction of steroid therapy led to sarcoidosis remission.

### Lupus proliferative glomerulonephritis

Two patients with previous history of steroid-resistant sarcoidosis had biopsy-proven lupus (SLE) glomerulonephritis (class IIIA in one case and IIIA-V in the second case). These patients developed sarcoidosis ten months and seven years, respectively, before the SLE diagnosis. Renal biopsies were performed because of significant proteinuria (1 g/d and 0.45 g/d, respectively) associated with microscopic hematuria. Antinuclear antibodies were not detected at the time sarcoidosis was diagnosed but became evident when the renal manifestations began. Therapy for lupus nephritis consisted of steroid and cyclophosphamide drugs in both cases and led to complete remission in one case, but failed to induce remission of lupus nephritis in the second case.

### Long-term clinical follow-up

Steroid and/or cytotoxic therapy was used to treat glomerular disease in 18 of 26 patients. Supportive treatment was initiated in eight patients (five patients with MN, two patients with IgAN and one patient with FSGS). At the end of follow-up (average of 8.4 years), complete remission of glomerular disease was confirmed in nine patients, whereas remission was considered to be partial for eight additional patients. Three other patients displayed significant persistent proteinuria. Six patients developed end-stage renal disease requiring intermittent hemodialysis and three of them underwent kidney transplantation. At the end of the follow-up, the mean GFR after excluding the six patients with end stage renal disease was estimated to be 67.8 mL/min per 1.73 m^2^ and three patients died.

## Discussion

In some sarcoidosis patients, the kidneys may be major sites of inflammation and granuloma formation, which leads to significant renal impairment [7]. In such cases, it is likely that infiltration of activated T cells and macrophages into the interstitium results in granuloma formation and interstitial nephritis. Alternatively, in rare cases of glomerular injury in the context of systemic sarcoidosis, the underlying molecular mechanisms of this association remain unclear. The aim of this retrospective was not to determine the incidence and/or the prevalence of glomerular involvement compared to interstitial nephritis related to sarcoidosis but exclusively to describe the spectrum of glomerular lesions observed in patients with sarcoidosis.

Our study demonstrated that a wide spectrum of glomerular lesions may be found in sarcoidosis patients. As these glomerular lesions do not share common pathophysiological mechanisms, the existence of a direct molecular link between sarcoidosis and glomerular pathology remains uncertain. Nevertheless, the close temporal relationship exhibited by nine patients, strongly suggests that glomerular disease and sarcoidosis may be linked in these patients.

Sarcoidosis is characterized by an infiltration of Th1 cells and macrophages into sites of inflammation and by a dominant expression of Th1 cytokines with low levels of Th2 cytokines [38,39]. Recent studies showed that sarcoidosis is associated with an expansion of a regulatory T cell subset with antiproliferative activity [40]. Most glomerular diseases found in patients with sarcoidosis are thought to be caused by functional disorders of the immune system. Compelling evidence suggests that primary MCNS and FSGS with relapse also result from perturbations of immune system [41]. Thus, we can postulate that cytokines produced locally by granulomas may contribute to alterations in the glomerular filtration barrier. Indeed, increased production of TNF-alpha by granuloma macrophages and activated T cells has been reported [42]. Experimental observations suggest that TNF-alpha may increase the permeability of the glomerular filtration barrier [43]. In accord with previous observations, we found that MN was the most frequent glomerular disease associated with sarcoidosis [6,10] and often preceded the sarcoidosis diagnosis. Sex ratio, mean age, and proportion of patients with nephrotic syndrome in patients with MN associated with sarcoidosis seem to be similar to those in previous series of patients with idiopathic membranous nephropathy [44-47]. The molecular link between these two conditions remains unclear since no target antigen or specific antibodies have yet been identified. Circulating immune complexes and hypergammaglobulinemia are a common finding in sarcoidosis patients [27]. Recently, Knehtl et al. described a patient with sarcoidosis and MN associated with anti-phospholipase A2 receptor antibodies (anti-PLA2R), suggesting that primary and secondary MN may share certain pathophysiological processes [14]. Three patients displayed IgAN simultaneously with active sarcoidosis. This close temporal relationship strongly suggests that immune dysregulation in sarcoidosis may lead to IgA deposition in the glomerular mesangium. Indeed, cytokine production may also play a critical role in the production and glycosylation of systemic IgA leading to IgAN [48]. The coexistence of SLE and sarcoidosis seems to be a very rare finding [49] even if a recent study suggests an increase of prevalence of SLE among sarcoidosis patients [50]. Weinberg et al. showed that the presence of anti-DNA antibodies in the serum of sarcoidosis patients did not predict the subsequent development of SLE [51]. We described here two interesting cases of lupus nephritis occurring in steroid-resistant sarcoidosis. In these two patients, we cannot exclude the possibility of a fortuitous association without known pathophysiologic relationship between both conditions. Nevertheless, these two cases emphasize the need to systematically screen sarcoidosis patients with renal impairment for SLE antibodies.

Mahevas et al. have previously reported that GTIN lesions occurred simultaneously with the first clinical manifestations of sarcoidosis in 81% of these cases [8]. In our study, only nine patients (35%) displayed biopsy-proven glomerular disease occurring simultaneously with sarcoidosis. Our study demonstrated that all types of glomerulopathies, except lupus nephritis, may be identified in patients with a first relapse of sarcoidosis. Among 11 patients with sarcoidosis that preceded the diagnosis of glomerular disease, none displayed sarcoidosis relapse at the time of renal biopsy. Nevertheless, three patients had untreated persistent sarcoidosis and two others presented steroid-resistant or steroid-dependent sarcoidosis. These five additional cases suggest that persistent granuloma activity may secondarily promote the development of glomerular disease.

Regardless of the types of glomerular lesions found in kidney biopsies, one major issue from our study was to analyze the response to steroid therapy in patients with glomerular diseases occurring simultaneously with sarcoidosis. A recent survey conducted in 47 patients showed that two criteria (the initial fibrotic score at renal biopsy and the response after one month of steroid therapy) were significantly associated with the renal prognosis of patients with GTIN related to sarcoidosis [8]. In our study, exclusive steroid therapy was administered to eight of the nine patients with simultaneous glomerular disease and sarcoidosis. Complete remission of both diseases occurred in a single case (pt7) whereas partial remission of the glomerulopathy and complete disappearance of sarcoidosis related symptoms occurred in three patients (pt5, pt8 and pt9). Despite steroid treatment, one patient (pt 4) displayed both persistent sarcoidosis and significant proteinuria. Finally, we found that sarcoidosis was frequently resistant to or dependent on steroid treatment (4/8 patients). However, the failure of steroid therapy to control sarcoidosis symptoms did not preclude the remission of glomerular disease in three patients (pt1, pt 3 and pt6). These observations are different from those of existing case reports, in which glomerular disease activity appeared to be influenced by the response of the sarcoidosis to steroid therapy [17,20,24,52]. Altogether, these observations show that the steroid responsiveness of sarcoidosis does not correlate with that of glomerular disease and suggest that both diseases require possibly a more aggressive therapy. Indeed, with a mean follow-up of 101 months, six of the 26 patients (23%) had chronic renal impairment requiring intermittent hemodialysis and/or renal transplantation, although their mean GFRs at the time of renal biopsy were not significantly impaired. In contrast, among patients with isolated GTIN who were followed for a median of 84 months, Rajakariar et al. demonstrated that exclusive steroid treatment may significantly improve GFR [9]. Similar results were found by Mahevas et al., suggesting that glomerular lesions are less steroid-sensitive than interstitial injury [8].

## Conclusion

Our study showed that the spectrum of glomerular lesions observed in the context of sarcoidosis is heterogeneous and that only 35% of the patients displayed both diseases simultaneously. For these cases, our study suggests that exclusive steroid therapy is less efficient that previously reported. Therefore, optimal therapeutic management can only be determined after further investigations.

## Competing interests

The authors of this manuscript declare that they have no competing interests.

## Authors' contributions

TS VA designed the study, analyzed and interpreted the data and wrote the manuscript, PL, DS reviewed the data and the paper, DJ, PV, JJB, PR, LM, MH, PG, GC,FV, JV, DD, FW, MM collected, reviewed data and contributed to identify patients who meet inclusion criteria, All authors approved the final version of the manuscript.

## References

[B1] Baughman RP Lower EE du Bois RM Sarcoidosis Lancet 2003 361 9363 1111 1118 10.1016/S0140-6736(03)12888-7 12672326

[B2] Iannuzzi MC Rybicki BA Teirstein AS Sarcoidosis N Eng J Med 2007 357 21 2153 2165 10.1056/NEJMra071714 18032765

[B3] Baughman RP Teirstein AS Judson MA Rossman MD Yeager H Jr Bresnitz EA DePalo L Hunninghake G Iannuzzi MC Johns CJ McLennan G Moller DR Newman LS Rabin DL Rose C Rybicki B Weinberger SE Terrin ML Knatterud GL Cherniak R Case Control Etiologic Study of Sarcoidosis research g: **Clinical characteristics of patients in a case control study of sarcoidosis** Am J Respir Crit Care Med 2001 164 10 Pt 1 1885 1889 11734441 10.1164/ajrccm.164.10.2104046

[B4] Hunninghake GW Crystal RG Pulmonary sarcoidosis: a disorder mediated by excess helper T-lymphocyte activity at sites of disease activity N Eng J Med 1981 305 8 429 434 10.1056/NEJM198108203050804 6454846

[B5] Nunes H Bouvry D Soler P Valeyre D Sarcoidosis Orphanet J Rare Dis 2007 2 46 10.1186/1750-1172-2-46 18021432 PMC2169207

[B6] Berliner AR Haas M Choi MJ Sarcoidosis: the nephrologist's perspective Am J Kidney Dis 2006 48 5 856 870 10.1053/j.ajkd.2006.07.022 17060009

[B7] Gobel U Kettritz R Schneider W Luft F The protean face of renal sarcoidosis Journal of the American Society of Nephrology: JASN 2001 12 3 616 623 11181812 10.1681/ASN.V123616

[B8] Mahevas M Lescure FX Boffa JJ Delastour V Belenfant X Chapelon C Cordonnier C Makdassi R Piette JC Naccache JM Cadranel J Duhaut P Choukroun G Ducroix JP Valeyre D Renal sarcoidosis: clinical, laboratory, and histologic presentation and outcome in 47 patients Medicine (Baltimore) 2009 88 2 98 106 10.1097/MD.0b013e31819de50f 19282700

[B9] Rajakariar R Sharples EJ Raftery MJ Sheaff M Yaqoob MM Sarcoid tubulo-interstitial nephritis: long-term outcome and response to corticosteroid therapy Kidney Int 2006 70 1 165 169 10.1038/sj.ki.5001512 16688117

[B10] Taylor RG Fisher C Hoffbrand BI Sarcoidosis and membranous glomerulonephritis: a significant association Br Med J (Clin Res Ed) 1982 284 6325 1297 1298 10.1136/bmj.284.6325.1297 PMC1498173 6803947

[B11] Oliver Rotellar JA Garcia Ruiz C Martinez Vea A Response to prednisone in membranous nephropathy associated with sarcoidosis Nephron 1990 54 2 195 10.1159/000185851 2314534

[B12] Toda T Kimoto S Nishio Y Ehara T Sasaki S Sarcoidosis with membranous nephropathy and granulomatous interstitial nephritis Intern Med 1999 38 11 882 886 10.2169/internalmedicine.38.882 10563750

[B13] Kaaroud H Fatma LB Beji S Jeribi A Maiz HB Moussa FB Goucha R Turki S Kheder A **Interstitial and glomerular renal involvement in sarcoidosis** Saudi J Kidney Dis Transpl 2008 19 1 67 71 18087126

[B14] Knehtl M Debiec H Kamgang P Callard P Cadranel J Ronco P Boffa JJ A case of phospholipase A(2) receptor-positive membranous nephropathy preceding sarcoid-associated granulomatous tubulointerstitial nephritis Am J Kidney Dis 2011 57 1 140 143 10.1053/j.ajkd.2010.09.015 21087816

[B15] Parry RG Falk C Minimal-change disease in association with sarcoidosis Nephrology, dialysis, transplantation: official publication of the European Dialysis and Transplant Association - European Renal Association 1997 12 10 2159 2160 10.1093/ndt/12.10.2159 9351083

[B16] Mundlein E Greten T Ritz E Graves' disease and sarcoidosis in a patient with minimal-change glomerulonephritis Nephrology, dialysis, transplantation: official publication of the European Dialysis and Transplant Association - European Renal Association 1996 11 5 860 862 10.1093/oxfordjournals.ndt.a027415 8671911

[B17] Nishimoto A Tomiyoshi Y Sakemi T Kanegae F Nakamura M Ikeda Y Shimazu K Yonemitsu N Simultaneous occurrence of minimal change glomerular disease, sarcoidosis and Hashimoto's thyroiditis Am J Nephrol 2000 20 5 425 428 10.1159/000013621 11093004

[B18] Lee SM Michael AF Focal glomerular sclerosis and sarcoidosis Arch Pathol Lab Med 1978 102 11 572 575 581450

[B19] Veronese FJ Henn Lde A Faccin CS Mussatto AV Paiva Neto A Edelweiss MI Morales JV Pulmonary sarcoidosis and focal segmental glomerulosclerosis: case report and renal transplant follow-up Nephrology, dialysis, transplantation: official publication of the European Dialysis and Transplant Association - European Renal Association 1998 13 2 493 495 10.1093/oxfordjournals.ndt.a027855 9509472

[B20] Peces R de la Torre M Sanchez-Fructuoso A Escalada P Focal segmental glomerulosclerosis associated with pulmonary sarcoidosis Nephron 1993 65 4 656 657 10.1159/000187591 8302436

[B21] Murray FE Lombard MG Donohoe JF Doyle GD Campbell E Alton BG Simultaneous presentation of IgA nephropathy and sarcoidosis Sarcoidosis 1987 4 2 134 136 3659616

[B22] Chung-Park M Lam M Yazdy AM IgA nephropathy associated with sarcoidosis Am J Kidney Dis 1990 15 6 601 602 2368701 10.1016/s0272-6386(12)80534-8

[B23] Tateno S Kobayashi Y Kobayashi F A case of sarcoidosis revealed in the course of IgA nephropathy Pathol Int 1994 44 5 387 390 8044308 10.1111/j.1440-1827.1994.tb02939.x

[B24] Taylor JE Ansell ID Steroid-sensitive nephrotic syndrome and renal impairment in a patient with sarcoidosis and IgA nephropathy Nephrology, dialysis, transplantation: official publication of the European Dialysis and Transplant Association - European Renal Association 1996 11 2 355 356 10.1093/oxfordjournals.ndt.a027267 8671793

[B25] Howard RS Gabriel R Glomerulonephritis in sarcoidosis: causal relationship unproven Postgrad Med J 1992 68 797 206 208 10.1136/pgmj.68.797.206 1589380 PMC2399239

[B26] Hagiwara S Ohi H Eishi Y Kodama F Tashiro K Makita Y Suzuki Y Maeda K Fukui M Horikoshi S Tomino Y A case of renal sarcoidosis with complement activation via the lectin pathway Am J Kidney Dis 2005 45 3 580 587 10.1053/j.ajkd.2004.11.020 15754281

[B27] Statement on sarcoidosis Joint Statement of the American Thoracic Society (ATS), the European Respiratory Society (ERS) and the World Association of Sarcoidosis and Other Granulomatous Disorders (WASOG) adopted by the ATS Board of Directors and by the ERS Executive Committee, February 1999 Am J Respir Crit Care Med 1999 160 2 736 755 10430755 10.1164/ajrccm.160.2.ats4-99

[B28] Scadding JG Prognosis of intrathoracic sarcoidosis in England. A review of 136 cases after five years' observation Br Med J 1961 2 5261 1165 1172 10.1136/bmj.2.5261.1165 14497750 PMC1970202

[B29] Levey AS Eckardt KU Tsukamoto Y Levin A Coresh J Rossert J De Zeeuw D Hostetter TH Lameire N Eknoyan G Definition and classification of chronic kidney disease: a position statement from Kidney Disease: Improving Global Outcomes (KDIGO) Kidney Int 2005 67 6 2089 2100 10.1111/j.1523-1755.2005.00365.x 15882252

[B30] Audard V Larousserie F Grimbert P Abtahi M Sotto JJ Delmer A Boue F Nochy D Brousse N Delarue R Remy P Ronco P Sahali D Lang P Hermine O Minimal change nephrotic syndrome and classical Hodgkin's lymphoma: report of 21 cases and review of the literature Kidney Int 2006 69 12 2251 2260 10.1038/sj.ki.5000341 16672913

[B31] D'Agati VD Fogo AB Bruijn JA Jennette JC Pathologic classification of focal segmental glomerulosclerosis: a working proposal Am J Kidney Dis 2004 43 2 368 382 10.1053/j.ajkd.2003.10.024 14750104

[B32] Glassock RJ Human idiopathic membranous nephropathy–a mystery solved? N Eng J Med 2009 361 1 81 83 10.1056/NEJMe0903343 19571287

[B33] Cattran DC Coppo R Cook HT Feehally J Roberts IS Troyanov S Alpers CE Amore A Barratt J Berthoux F Bonsib S Bruijn JA D'Agati V D'Amico G Emancipator S Emma F Ferrario F Fervenza FC Florquin S Fogo A Geddes CC Groene HJ Haas M Herzenberg AM Hill PA Hogg RJ Hsu SI Jennette JC Joh K Julian BA Kawamura T Lai FM Leung CB Li LS Li PK Liu ZH Mackinnon B Mezzano S Schena FP Tomino Y Walker PD Wang H Weening JJ Yoshikawa N Zhang H Working Group of the International Ig ANN, the Renal Pathology S The Oxford classification of IgA nephropathy: rationale, clinicopathological correlations, and classification Kidney Int 2009 76 5 534 545 10.1038/ki.2009.243 19571791

[B34] Hochberg MC Updating the American College of Rheumatology revised criteria for the classification of systemic lupus erythematosus Arthritis Rheum 1997 40 9 1725 9324032 10.1002/art.1780400928

[B35] Weening JJ D'Agati VD Schwartz MM Seshan SV Alpers CE Appel GB Balow JE Bruijn JA Cook T Ferrario F Fogo AB Ginzler EM Hebert L Hill G Hill P Jennette JC Kong NC Lesavre P Lockshin M Looi LM Makino H Moura LA Nagata M The classification of glomerulonephritis in systemic lupus erythematosus revisited Journal of the American Society of Nephrology: JASN 2004 15 2 241 250 10.1097/01.ASN.0000108969.21691.5D 14747370

[B36] Schena FP Survey of the Italian Registry of Renal Biopsies. Frequency of the renal diseases for 7 consecutive years. The Italian Group of Renal Immunopathology Nephrology, dialysis, transplantation: official publication of the European Dialysis and Transplant Association - European Renal Association 1997 12 3 418 426 10.1093/ndt/12.3.418 9075118

[B37] Swaminathan S Leung N Lager DJ Melton LJ 3rd Bergstralh EJ Rohlinger A Fervenza FC Changing incidence of glomerular disease in Olmsted County, Minnesota: a 30-year renal biopsy study Clinical journal of the American Society of Nephrology: CJASN 2006 1 3 483 487 10.2215/CJN.00710805 17699249

[B38] Konishi K Moller DR Saltini C Kirby M Crystal RG Spontaneous expression of the interleukin 2 receptor gene and presence of functional interleukin 2 receptors on T lymphocytes in the blood of individuals with active pulmonary sarcoidosis J Clin Invest 1988 82 3 775 781 10.1172/JCI113678 3138285 PMC303582

[B39] Bargagli E Mazzi A Rottoli P Markers of inflammation in sarcoidosis: blood, urine, BAL, sputum, and exhaled gas Clin Chest Med 2008 29 3 445 458 viii 10.1016/j.ccm.2008.03.004 18539237

[B40] Miyara M Amoura Z Parizot C Badoual C Dorgham K Trad S Kambouchner M Valeyre D Chapelon-Abric C Debre P Piette JC Gorochov G The immune paradox of sarcoidosis and regulatory T cells J Exp Med 2006 203 2 359 370 10.1084/jem.20050648 16432251 PMC2118208

[B41] Zhang S Audard V Fan Q Pawlak A Lang P Sahali D Immunopathogenesis of idiopathic nephrotic syndrome Contrib Nephrol 2011 169 94 106 21252513 10.1159/000313947

[B42] Agostini C Adami F Semenzato G New pathogenetic insights into the sarcoid granuloma Curr Opin Rheumatol 2000 12 1 71 76 10.1097/00002281-200001000-00012 10647958

[B43] Laflam PF Garin EH Effect of tumor necrosis factor alpha and vascular permeability growth factor on albuminuria in rats Pediatr Nephrol 2006 21 2 177 181 10.1007/s00467-005-2078-3 16211409

[B44] Noel LH Zanetti M Droz D Barbanel C Long-term prognosis of idiopathic membranous glomerulonephritis. Study of 116 untreated patients Am J Med 1979 66 1 82 90 10.1016/0002-9343(79)90486-8 420255

[B45] Schieppati A Mosconi L Perna A Mecca G Bertani T Garattini S Remuzzi G Prognosis of untreated patients with idiopathic membranous nephropathy N Eng J Med 1993 329 2 85 89 10.1056/NEJM199307083290203 8510707

[B46] Ponticelli C Zucchelli P Passerini P Cesana B Locatelli F Pasquali S Sasdelli M Redaelli B Grassi C Pozzi C A 10-year follow-up of a randomized study with methylprednisolone and chlorambucil in membranous nephropathy Kidney Int 1995 48 5 1600 1604 10.1038/ki.1995.453 8544420

[B47] Jha V Ganguli A Saha TK Kohli HS Sud K Gupta KL Joshi K Sakhuja V A randomized, controlled trial of steroids and cyclophosphamide in adults with nephrotic syndrome caused by idiopathic membranous nephropathy Journal of the American Society of Nephrology: JASN 2007 18 6 1899 1904 10.1681/ASN.2007020166 17494881

[B48] Barratt J Smith AC Molyneux K Feehally J Immunopathogenesis of IgAN Semin Immunopathol 2007 29 4 427 443 10.1007/s00281-007-0089-9 17851660

[B49] Wesemann DR Costenbader KH Coblyn JS Co-existing sarcoidosis, systemic lupus erythematosus and the antiphospholipid antibody syndrome: case reports and discussion from the Brigham and Women's Hospital Lupus Center Lupus 2009 18 3 202 205 10.1177/0961203308100483 19213857

[B50] Rajoriya N Wotton CJ Yeates DG Travis SP Goldacre MJ Immune-mediated and chronic inflammatory disease in people with sarcoidosis: disease associations in a large UK database Postgrad Med J 2009 85 1003 233 237 10.1136/pgmj.2008.067769 19520873

[B51] Weinberg I Vasiliev L Gotsman I Anti-dsDNA antibodies in sarcoidosis Semin Arthritis Rheum 2000 29 5 328 331 10.1016/S0049-0172(00)80019-0 10805357

[B52] Nishiki M Murakami Y Yamane Y Kato Y Steroid-sensitive nephrotic syndrome, sarcoidosis and thyroiditis–a new syndrome? Nephrology, dialysis, transplantation: official publication of the European Dialysis and Transplant Association - European Renal Association 1999 14 8 2008 2010 10.1093/ndt/14.8.2008 10462286

